# Impact of metabolic syndrome on the viability of human spermatozoa: a cross-sectional descriptive study in men from infertile couples

**DOI:** 10.1186/s12610-021-00142-8

**Published:** 2021-10-07

**Authors:** Minh Tam Le, Hiep Tuyet Thi Nguyen, Hong Nhan Thi Dang, Thai Thanh Thi Nguyen, Trung Van Nguyen, Quoc Huy Vu Nguyen

**Affiliations:** 1 Center for Reproductive Endocrinology and Infertility, Hue University of Medicine and Pharmacy, Hue University, 06 Ngo Quyen Street, Hue City, Hue, Vietnam; 2 Department of Obstetrics and Gynecology, Hue University of Medicine and Pharmacy, Hue University, Hue, Vietnam

**Keywords:** Spermatozoa, Survival test, Infertility, Metabolic syndrome, Spermatozoïdes, test de survie, infécondité, syndrome métabolique

## Abstract

**Background:**

A direct association between metabolic syndrome (MetS) and sperm production/function has been proposed. In this cross-sectional study, we aimed to determine the impact of MetS on sperm survival. Men from infertile couples treated at Hue University Hospital, Vietnam, were enrolled in this study, which spanned the October 2018 to October 2020 period. The general characteristics of the patients, including body mass index (BMI), waist-to-hip ratio (WHR), the levels of different biochemicals, and semen parameters were determined, and sperm survival tests (SSTs) were performed. The modified National Cholesterol Education Program (NCEP) Adult Treatment Panel (ATP) III for the Asian population was used for MetS diagnosis.

**Results:**

Men with an abnormal waist circumference (≥ 90 cm) showed a higher rate of abnormal SST results (30.1% vs. 16.7%, *p* = 0.012). The frequency of abnormal SST results in patients with MetS (72.3%) was significantly higher than that in individuals without MetS (53.4%) (*p* = 0.02). Furthermore, the percentage of abnormal SST results in patients with MetS and with BMI ≥ 23 was significantly higher than those in individuals without MetS (77.1% vs. 55.2%, *p* = 0.03). Weak negative correlations were also observed between the patients’ age and the SST results.

**Conclusion:**

Sperm viability was lower in men with MetS. We also observed that age and BMI were independent factors associated with abnormal SST.

## Résumé

**Contexte:**

Une association directe entre le syndrome métabolique (MetS) et la production/fonction des spermatozoïdes a été proposée. Dans cette étude transversale, nous avons cherché à déterminer l’impact du MetS sur le taux de survie des spermatozoïdes (TSS). Des hommes issus de couples inféconds traités à l’hôpital universitaire de Hue, au Vietnam, ont été inclus dans cette étude qui s’est. étendue sur la période d’octobre 2018 à octobre 2020. Les caractéristiques générales des patients, y compris l’indice de masse corporelle (IMC), le rapport taille-hanche, les niveaux de différents paramètres biochimiques et les paramètres de sperme ont été déterminés, ainsi que les taux de survie des spermatozoïdes (TSS). Le panel modifié du traitement des adultes (ATP III) du Programme National d’Education sur le Cholestérol (NCEP) pour la population asiatique a été utilisé pour le diagnostic de MetS.

**Résultats:**

Les hommes avec un tour de taille anormal (≥ 90 cm) ont montré un taux plus élevé de résultats anormaux de SST (30,1% versus 16,7%, *p* = 0,012). La fréquence de résultats anormaux de TSS chez les patients avec MetS (72.3%) était significativement plus élevée que celle des individus sans MetS (53,4%) (*p* = 0,02). En outre, le pourcentage de résultats anormaux de TSS chez les patients avec MetS et avec un BMI ≥ 23 était significativement plus élevé que celui des individus sans MetS (77,1% versus 55,2%, *p* = 0,03). On a également observé de faibles corrélations négatives entre l’âge des patients et les résultats du TSS.

**Conclusion:**

La viabilité des spermatozoïdes était plus faible chez les hommes atteints de MetS. Nous avons également observé que l’âge et l’IMC étaient des facteurs indépendants associés à un TSS anormal.

## Background

Metabolic syndrome (MetS) constitutes a group of abnormalities, including obesity, dyslipidemia, hypertension, and insulin resistance [1]. According to the US National Cholesterol Education Adult Treatment Panel III (NCEP ATP III), MetS in men is diagnosed in individuals showing more than three of the following criteria: waist circumference ≥ 102 cm, blood pressure ≥ 130/85 mmHg, HDL < 40 mg/dL, triglycerides ≥150 mg/dL, and fasting glucose ≥100 mg/dL [2]; Notably, the ATP III waist circumference (WC) criterion has been adjusted for the Asian population to ≥ 90 cm in men [3]. Chronic inflammation is considered as an aetiological and mechanistic phenomenon in MetS. It is well known that inflammatory cytokines disrupt the hypothalamic-pituitary-testes axis; therefore, it is not unreasonable to consider that MetS can impact on (male) fertility [1].

Male fertility is often determined based on the quality of spermatozoa. In this regard, the sperm survival test (SST), which was first mentioned in a study by Fuse in 1990, has as objective to establish a new screening test for sperm fertility potential, has been suggested. In the previous study, sperm motility is examined after 6, 12, 24, 36, and 48 h of incubation at temperature of 37 °C [4]. Although, SST was originally designed to evaluate sperm viability, sperm motility (surely these motile sperm are viable) plays a more important role in fertilization given that it determines the forward movement of the spermatozoa in the female genital tract. Thus, the SST results are considered positive when motile swim-up spermatozoa are observed at the indicated times. Additionally, the SST results are considered normal when the ratio of progressing spermatozoa after 24 h of incubation per initial density of progressing spermatozoa is ≥ 50% [5, 6]. In line with these results, the efficacy of the SST in in-vitro fertilization (IVF) was estimated to be 0.71, which is better than that corresponding to the conventional sperm fertilization method. Therefore, the SST is considered useful in the prediction of fertilization (and male fertility) [5]. In fact, abnormal SST may bring about as much as a 90% decrease in the chances of realizing successful conventional IVF cycles. Further, normal SST results can predict the IVF results with a sensitivity of 87%, a specificity of 65%, and a positive predictive value of 90% (considering the male factor only) [7]. Presently, many more studies have reported the predictive power of SST after 24 h based on fertilization outcomes [8, 9], highlighting this test as a clear indicator of male fertility.

In several studies, direct associations between MetS and sperm production as well as sperm function have been suggested [10]. For instance, MetS is said to be associated with a lower testosterone level and with the presence of hypogonadism, abnormal sperm morphology, and erectile dysfunction in men of infertile couples; however, it did not induce any changes in gonadotropin concentrations [11]. Additionally, MetS is associated with low semen volume, low sperm count, and abnormal sperm morphology [12]. Leisegang et al. demonstrated that MetS is associated with a decrease in total sperm count, sperm motility, sperm vitality, mitochondrial membrane potential, and free testosterone, and an increased DNA fragmentation index [13]. Moskovtsev et al. reported that sperm DNA fragmentation is negatively correlated with progressive sperm motility, concentration, normal morphology, motility, and survival index [6]. However, some studies have reported that MetS seems to be unrelated to semen quality [14, 15]. For instance, age may have been a confounding factor, since age shows a significant positive correlation with sperm DNA fragmentation and a negative correlation with sperm concentration, and viability [6]. Therefore, the correlation between MetS and reduced sperm parameters remains unclear. This cross-sectional descriptive study aimed to clarify the association between MetS and sperm survival.

## Methods

### Study design

This cross-sectional descriptive study was performed at the Hue Center for Reproductive Endocrinology and Infertility (HueCREI), Hue University Hospital, Vietnam, from October 2018 to October 2020. The selection criteria in this study included men of infertile couples according to WHO (2010) standards [16]; only men that accepted to be subjected to anthropometry, biochemical assays, and semen analysis were enrolled. Men with acute urinary tract infection, malignant diseases, retrograde ejaculation or azoospermia were excluded; sperm samples retrieved via surgery were also not used. All the participants were examined in accordance with usual clinical checkup. Thus, patient-related information, including age, reproductive history, and history of andrological pathologies such as cryptorchidism and infectious diseases such as orchitis and epididymitis was collected. The patients were also examined to check the presence of two testicles into the scrotum, the presence of a varicocele of the two vasa differentia, and testicular size or volume. Anthropometric characteristics, including height, weight, waist circumference, hip circumference, and blood pressure, were recorded. Biochemical tests were also performed to determine fasting plasma glucose and blood lipid levels. Additionally, semen analysis was also performed from all the participants. This study was approved by the Ethics Committee of Hue University of Medicine and Pharmacy (Approval number H2019/436).

The sample size was calculated according to the following formula: $n={Z}_{\alpha /2}^2\frac{p\left(1-p\right)}{\Delta ^2}$; Z_α/2_ was defined as 1.96 for a confidence level (α) of 95%. The prevalence of MetS in Vietnamese men (p) has been previously reported as 14.8% [17]. Additionally, Δ was defined as 0.05 and the calculated minimum sample size to ensure statistical power was 194. Therefore, a total of 195 men were enrolled in the study.

### Data collection

The body weight of the participants was measured using a SECA scale (SECA, France), and their heights were measured in the standing position using a three-piece wooden ruler. The body mass index (BMI) was then calculated as the weight in kilograms divided by the square of height in meters (kg/m^2^); Thereafter, the participants were categorized in accordance with the Asian population criteria: underweight (< 18.5 kg/m^2^), normal weight (18.5–22.9 kg/m^2^), overweight (23–24.9 kg/m^2^), and obese (≥ 25 kg/m^2^) [18, 19]. Waist circumference (WC) was measured midway between the lower limit of the rib cage and the iliac crest, and the hip circumference was measured as the maximum circumference of the buttocks. The waist-to-hip ratios (WHRs) of the participants were then determined; a WHR above 0.9 was classified as central obesity based on the Asian-Pacific specific and WHO recommendations [20]. Blood pressure was measured on the upper right arm after a 15 min rest using a standard mercury sphygmomanometer (ALPK2, Japan).

Additionally, venous blood samples were collected in the morning after overnight fasting, and the levels of glucose, lipids, including total cholesterol, triglycerides (TG), high- (HDL-C) and low-density lipoprotein cholesterol (LDL-C) were determined using the Roche/Hitachi Cobas C system (Module COBAS 4000/6000, Roche Diagnostics, Indianapolis, IN, USA), according to the manufacturer’s instructions. All the measurements were performed at the Laboratory Center of Hue University Hospital.

The modified NCEP ATP III criteria, adapted for Asian individuals, were used to define MetS. A diagnosis of MetS was confirmed when at least three of the following criteria were present: WC ≥ 90 cm, TG ≥ 1.7 mmol/L, HDL-C < 1.03 mmol/L, blood pressure ≥ 130/85 mmHg, and fasting glucose ≥5.6 mmol/L [3].

#### Semen analysis

Semen samples were obtained via masturbation after 3–7 days of sexual abstinence and thereafter, analyzed following WHO 2010 standards [16]. Sperm motility was analyzed manually via phase-contrast microscopy (Primo Star, Zeiss, Germany) at 400X magnification, while sperm vitality was assessed by eosin staining. Two hundred cells were counted immediately after the liquefaction of the semen samples. For morphological assessment, after staining with Giemsa, the morphology of the sperm head, sperm neck, midpiece, and tail, as well as the cytoplasmic droplets were determined under a microscope at 1000X magnification.

### Sperm survival test (SST)

Semen samples were collected after 3–7 days of abstinence by masturbation. After incubation for 30 min to allow liquefaction, samples were then introduced above the density gradient of a Sil-select Plus media (Fertipro, Beernem, Belgium), consisting of 1.5 ml of the upper layer (45%) and 1.5 ml of the lower layer (90%). Thereafter, the mixture was centrifuged at 350 g for 15 min. Sperm samples were then washed twice with 3 mL Ferticult Flushing (FertiPro, Beernem, Belgium) and centrifuged for 10 min at 350 g. The final pellet was resuspended in 0.5 ml Ferticult Flushing medium. Sperm motility and vitality were then evaluated immediately after the last washing step, as well as after 24 h of incubation at 37 °C.

The percentage of sperm motility was evaluated by counting moving and non-moving spermatozoa. The percentage of sperm vitality (expressed as %) was assessed using the eosin staining technique. Totally, the number of stained (dead) and unstained (vital) cells were determined with the aid of a laboratory counter. At least 200 spermatozoa were counted in each test by two experienced embryologists. The SST results were then evaluated as percentages of motile and viable spermatozoa at 24 h and the sperm motility index (SMI) as well as the sperm vitality index (SVI) were calculated as follows [6]:

$$ \mathrm{SMI}\ \left(\%\right)=\frac{\mathrm{percentage}\ \mathrm{of}\ \mathrm{motile}\ \mathrm{spermatozoa}\ \mathrm{after}\ 24\ \mathrm{h}\ \mathrm{incubation}}{\mathrm{Initial}\ \mathrm{percentge}\ \mathrm{of}\ \mathrm{mortile}\ \mathrm{spermatozoa}}\times 100 $$

$$ \mathrm{SVI}\ \left(\%\right)=\frac{\mathrm{percentage}\ \mathrm{of}\ \mathrm{viable}\ \mathrm{spermatozoa}\ \mathrm{after}\ 24\ \mathrm{h}\ \mathrm{incubation}}{\mathrm{Initial}\ \mathrm{percentge}\ \mathrm{of}\ \mathrm{viable}\ \mathrm{spermatozoa}}\times 100 $$

T results were considered normal when the SMI was ≥ 50% [5].

### Statistical analysis

Statistical analysis was performed using SPSS, v20.0 (IBM Corp, Armonk, NY, USA), and all the data obtained are presented as the mean ± standard deviation, or as percentages. Categorical data were assessed for normal distribution by performing the Shapiro–Wilk test. Further, Student’s t-test and Chi squared test were used to compare Gaussian data. The Mann-Whitney U-test was used to compare non-Gaussian data, where appropriate, for two-group comparisons. Statistical significance was set at *p* < 0.05. Additionally, to determine the correlations between the different variables based on correlation coefficients, Spearman correlations analysis was performed.

## Results

### Characteristics of the study population

Of the 195 men of infertile couples included in this study, the proportion with MetS was 24.1% (i.e. 47/195), as shown in Table 1. The mean age, mean BMI, WC and WHR were remarkably higher for subjects with MetS than for those without MetS. Based on the total number of participating infertility couples included in this study, infertility resulting from male-related factors, female factors, both male and female factors and unexplained causes accounted for 29.7, 39.4, 22.1, and 8.7% of all the infertility cases. In male related factors, history of mump infection, sexual transmitted diseases, urogenital surgery and varicoceles accounted for 9.7, 1, 2.6 and 5.6%, respectively. The proportion of these factors between MetS and non-MetS groups were not significant different. Regarding lipid profiles, individuals with MetS showed statistically significant higher mean concentrations of cholesterol, triglycerides, and LDL-C, and lower levels of HDL-C than individuals without MetS. Additionally, the fasting glucose levels in the MetS group were significantly higher than those in the non-MetS group, and no group differences with respect to blood pressure, alcohol consumption, or smoking habits were observed. The differences in the compositions of fresh semen samples from both groups were not statistically significant.

**Table 1:** General characteristics among men in infertile couples with and without metabolic syndrome

Characteristics	Total (***n*** = 195)	Metabolic syndrome		***P***-value^**a**^
		MetS (***n*** = 47)	Non-MetS (***n*** = 148)	
Age (years) (mean ± SD)	34.73 ± 5.51	36.21 ± 3.82	34.26 ± 5.88	0.03
Infertility type [n (%)]				
Primary	128 (65.6%)	31 (24.2%)	97 (75.8%)	0.85
Secondary	67 (34.4%)	16 (24.2%)	50 (75.8%)	
Male factor Infertility [n (%)]	101 (51,8%)	25 (53.2%)	76 (51.4%)	0.83
Mump	19 (9.7%)	5 (10.6%)	14 (9.5%)	0.78
Testicular infection	1 (0.5%)	1 (2.1%)	0 (0%)	0.24
*Sexually Transmitted Diseases*	2 (1.0%)	2 (4.3%)	0 (0%)	0.06
Urogenital surgery	5 (2.6%)	2 (4.3%)	3 (2.0%)	0.60
Varicocele	11 (5.6%)	5 (10.6%)	6 (4.1%)	0.14
Female factor infertility [n(%)]	120 (61.5%)	29 (61.7%)	91 (61.5%)	0.98
Unexplained infertility [n(%)]	17 (8.7%)	3 (6.4%)	14 (9.5%)	0.77
BMI (kg/m^2^) (mean ± SD)	22.93 ± 2.96	25.14 ± 3.01	22.22 ± 2.58	0.00
< 23 [n (%)]	102 (52.3%)	12 (11.8%)	90 (88.2%)	0.00
≥ 23 [n (%)]	93 (47.7%)	35 (37.6%)	58 (62.4%)	
WC (cm) (mean ± SD)	82.55 ± 8.60	71.21 ± 9.27	62.01 ± 7.81	0.00
WHR (mean ± SD)	0.87 ± 0.06	0.91 ± 0.07	0.86 ± 0.051	0.00
Blood pressure (mean ± SD)				
SBP, (mmHg)	112.64 ± 7.87	112.87 ± 7.85	112.57 ± 7.90	0.82
DBP, (mmHg)	71.33 ± 5.53	72.34 ± 6.24	71.01 ± 5.26	0.15
Lipid profile (mean ± SD)				
Cholesterol (mmol/L)	4.77 ± 0.92	5.3 ± 0.99	4.59 ± 0.83	0.00
Triglycerides (mmol/L)	2.21 ± 1.30	3.31 ± 1.15	1.86 ± 1.14	0.00
LDL-C (mmol/L)	3.22 ± 0.87	3.69 ± 0.86	3.07 ± 0.82	0.00
HDL-C (mmol/L)	1.22 ± 0.31	1.02 ± 0.23	1.29 ± 0.31	0.00
Fasting glucose (mmol/L) (mean ± SD)	5.47 ± 0.88	6.16 ± 1.18	5.25 ± 0.62	0.00
Alcohol consumption [n (%)]				
Yes	45 (23.1%)	10 (21.3%)	35 (23.6%)	0.74
No	150 (76.9%)	37 (78.7%)	113 (76.4%)	
Smoking [n (%)]				
Yes	34 (17.4%)	11 (23.4%)	23 (15.5%)	0.22
No	161 (82.6%)	36 (76.6%)	125 (84.5%)	
Fresh semen analysis (mean ± SD)				
pH	7.08 ± 0.26	7.11 ± 0.29	7.08 ± 0.25	0.80
Sexual abstinence (days)	4.20 ± 1.31	3.96 ± 1.25	4.28 ± 1.33	0.15
Semen volume (ml)	1.84 ± 0.95	1.90 ± 1.04	1.82 ± 0.93	0.70
Sperm count (× 10^6^/ml)	33.80 ± 12	35.89 ± 14.28	33.13 ± 14.62	0.26
Sperm vitality (%)	81.44 ± 7.63	82.62 ± 5.93	81.07 ± 8.08	0.27
Progressive motility (%)	33.26 ± 10.84	35.30 ± 9.61	32.61 ± 11.16	0.14
Normal sperm morphology (%)	3.89 ± 2.22	4.21 ± 2.33	3.79 ± 2.18	0.22

*BMI* body mass index; *DPB* diastolic blood pressure; *HDL-C* high density lipoprotein cholesterol; *LDL-C* low density lipoprotein cholesterol; *MetS* metabolic syndrome; *SBP* systolic blood pressure; *WC* waist circumference; *WHR* waist-to-hip ratio

Values were expressed as mean ± SD and number (percentage)

^a^ Comparison was performed between men with and without MetS using the independent-samples *t* test and the chi-square test for Gaussian data and the Mann-Whitney U-test was used to compare non-Gaussian data

As shown in Table 2, the proportion of men with abnormal SST results was 57.9%. Although the mean age of the participants in the abnormal SST group was slightly higher than that of the participants in the normal SST group, this difference was not statistically significant. Similarly, no significant differences were observed with respect to mean BMI or WHR. However, in patients with increased WC (≥ 90 cm), the proportion of abnormal SST cases was significantly higher (30.1% vs. 16.7%; *p* = 0.01). In fact, this was the only observed significant difference, individuals with normal and abnormal SST results showed no significant differences with respect to the levels of triglycerides, LDL-C, HDL-C, and fasting glucose. Further, apart from the fact that the abnormal SST group showed lower semen volume, fresh semen samples from individuals in both groups showed no significant differences.

**Table 2:** General characteristics among men in infertile couples with normal and abnormal sperm survival test

Characteristics	Total (***n*** = 195)	Sperm survival test		***P***-value^**a**^
		Abnormal SST (***n*** = 113)	Normal SST (***n*** = 82)	
Age (years) (mean ± SD)	34.73 ± 5.51	35.30 ± 5.65	33.95 ± 5.25	0.09
Infertility type [n (%)]				
Primary	128 (65.6%)	79 (69.9%)	49 (59.8%)	0.15
Secondary	67 (34.4%)	34 (30.1%)	33 (40.2%)	
BMI (kg/m^2^) (mean ± SD)	22.93 ± 2.96	23.24 ± 3.27	22.50 ± 2.42	0.47
< 23 [n (%)]	102 (52.3%)	54 (52.9%)	48 (47.1%)	0.15
≥ 23 [n (%)]	93 (47.7%)	59 (63.4%)	34 (36.6%)	
WC (cm) (mean ± SD)	82.55 ± 8.60	83.54 ± 8.91	81.18 ± 8.01	0.59
< 90 [n (%)]	149 (76.4%)	79 (69.9%)	70 (83.3%)	0.01
≥ 90 [n (%)]	46 (23.6%)	34 (30.1%)	12 (16.7%)	
WHR (mean ± SD)	0.87 ± 0.06	0.87 ± 0.06	0.86 ± 0.06	0.26
≤ 0.9 [n (%)]	142 (72.8%)	77 (54.2%)	65 (45.8%)	0.09
> 0.9 [n (%)]	53 (27.2%)	36 (67.9%)	17 (32.1%)	
Blood pressure (mean ± SD)				
SBP (mmHg) [n (%)]	112.64 ± 7.87	112.61 ± 8.69	112.68 ± 6.63	0.95
DBP (mmHg) [n (%)]	71.33 ± 5.53	71.32 ± 5.99	71.34 ± 4.84	0.98
Lipid profile (mean ± SD)				
Cholesterol (mmol/L)	4.77 ± 0.92	4.77 ± 0.90	4.76 ± 0.95	0.91
Triglycerides (mmol/L)	2.21 ± 1.30	2.35 ± 1.31	2.02 ± 1.27	0.09
LDL-C (mmol/L)	3.22 ± 0.87	3.24 ± 0.86	3.20 ± 0.89	0.73
HDL-C (mmol/L)	1.22 ± 0.31	1.20 ± 0.28	1.26 ± 0.34	0.22
Fasting glucose (mmol/L) (mean ± SD)	5.47 ± 0.88	5.54 ± 1.02	5.37 ± 0.63	0.16
Alcohol consumption [n (%)]				
Yes	45 (23.1%)	28 (62.2%)	17 (37.8%)	0.51
No	150 (76.9%)	85 (56.7%)	65 (43.3%)	
Smoking [n (%)]				
Yes	34 (17.4%)	23 (67.6%)	11 (32.4%)	0.21
No	161 (82.6%)	90 (55.9%)	71 (44.1%)	
Fresh semen analysis (mean ± SD)				
pH	7.08 ± 0.26	7.06 ± 0.23	7.11 ± 0.29	0.22
Sexual abstinence (days)	4.20 ± 1.31	4.21 ± 1.33	4.18 ± 1.29	0.88
Semen volume (ml)	1.84 ± 0.95	1.71 ± 0.88	2.02 ± 1.01	0.01
Sperm count (×10^6^/ml)	33.80 ± 12.	32.91 ± 15.37	35.01 ± 13.35	0.32
Sperm vitality (%)	81.44 ± 7.63	80.39 ± 8.88	82.89 ± 5.20	0.06
Progressive motility (%)	33.26 ± 10.84	32.64 ± 11.98	34.12 ± 9.04	0.33
Normal sperm morphology (%)	3.89 ± 2.22	3.76 ± 2.18	4.06 ± 2.27	0.28

*BMI* body mass index; *DPB* diastolic blood pressure; *HDL-C* high density lipoprotein cholesterol; *LDL-C* low density lipoprotein cholesterol; *SBP* systolic blood pressure; *SST* sperm survival test; *WC* waist circumference; *WHR* waist-to-hip ratio

^a^ Student’s t-test, Chi square test were used to compare Gaussian data and the Mann-Whitney U-test was used to compare non-Gaussian data. Values were expressed as mean ± SD and number (percentage)

### Relationship between sperm survival and MetS as well as a high BMI

The relationship between MetS and SST parameters, including sperm motility and viability at different time points is presented in Table 3. No statistically significant differences in progressive sperm motility and survival between individuals with MetS and those without MetS at 0 and 24 h were observed. In line with these results, the SMI and SVI values corresponding to the MetS and non-MetS groups at 24 h were not significantly different. However, the proportion of abnormal SST results in the MetS group was higher than that in the non-MetS group. We analysed the relationship between MetS and SST results based on the BMI group (Table 4). Interestingly, it was observed that for the group with high BMI (≥ 23), the frequency of abnormal SST in patients with MetS was significantly higher than that in patients without MetS. These differences were not detected in individuals with a BMI < 23.

**Table 3:** Sperm survival test in men with and without metabolic syndrome

SST	Total (***n*** = 195)	Metabolic syndrome		***P***-value^**a**^
		MetS (***n*** = 47)	Non-MetS (***n*** = 148)	
PR motility (%)				
PR-0 h	76.03 ± 10.52	76.53 ± 9.65	75.87 ± 10.81	0.71
PR-24 h	30.99 ± 15.80	28.81 ± 14.96	31.69 ± 16.05	0.28
Viability (%)				
Viable-0 h	86.78 ± 6.91	86.53 ± 7.04	86.86 ± 6.89	0.77
Viable-24 h	54.98 ± 17.33	53.15 ± 17.24	55.57 ± 17.38	0.41
Result of SST [n(%)]				
Normal	82 (42.1%)	13 (27.7%)	69 (46.6%)	0.02
Abnormal	113 (57.9%)	34 (72.3%)	79 (53.4%)	
SMI-24 h (%)	40.20 ± 18.60	36.87 ± 16.47	41.24 ± 19.16	0.16
SVI-24 h (%)	62.68 ± 17.34	60.79 ± 17.01	63.28 ± 17.57	0.39

*PR* progressive; *SMI* sperm motility index; *SST* sperm survival test; *SVI* sperm vitality index

Sperm parameters were assessed after prepared by gradient density centrifugation

Values were expressed as mean ± SD and the percentage

^a^ Comparison was performed between men with and without MetS using the independent-samples *t* test and the chi-square test

**Table 4:** Sperm survival test in men with and without metabolic syndrome classified in BMI groups

BMI	MetS subgroups	Abnormal SST	Normal SST	***P1***-value	***P2***-value
**BMI < 23**	Non-MetS (90, 88.2%)	47 (52.2%)	43 (47.8%)	0.690	0.02
	MetS (12, 11.8%)	7 (58.3%)	5 (41.7%)		
**BMI ≥ 23**	Non-MetS (58, 62.4%)	32 (55.2%)	26 (44.8%)	0.033	
	MetS (35, 37.6%)	27 (77.1%)	8 (22.9%)		

*BMI* body mass index; *MetS* metabolic syndrome; *SST* sperm survival test

Values were expressed as the number (percentage)

Comparison was performed between men with and without MetS (*P1*-value) and in BMI groups (*P2*-value) using the chi-square test

### Relationship between age and sperm survival

Correlations between sperm survival parameters and age, anthropometric findings, glucose levels, and the lipid profiles of the individuals enrolled in this study were analyzed (Table 5). Thus, a weak negative correlation was observed between age and SST result indicators, including progressive motility (PR) (r = − 0.17, *p* = 0.02), viability (r = − 0.18, *p* = 0.01), SMI (r = − 0.18, p = 0.01), and SVI (r = − 0.19, p = 0.01) at 24 h. However, the anthropometric findings, glucose levels, and lipid profile did not show any correlation with any of the SST result indicators.

**Table 5:** Correlation between age, the anthropometry, the components of MetS and sperm survival test

SST		Age	BMI	WC	WHR	Glucose	Cholesterol	Triglycerides	HDL-C	LDL-C
PR-0 h	r	−0.06	0.03	0.07	0.00	− 0.09	0.00	0.10	0.07	−0.07
	p	0.40	0.69	0.32	0.97	0.19	0.95	0.16	0.30	0.33
PR-24 h	r	−0.17^*^	−0.07	−0.06	− 0.04	−0.07	− 0.01	−0.04	0.10	−0.04
	p	0.02	0.30	0.39	0.55	0.32	0.95	0.57	0.15	0.57
SMI-24 h	r	−0.18^*^	−0.08	−0.09	− 0.06	−0.07	− 0.01	−0.06	0.09	−0.04
	p	0.01	0.25	0.23	0.42	0.31	0.90	0.37	0.20	0.62
Viability-0 h	r	−0.07	−0.04	0.02	−0.01	−0.09	− 0.03	0.05	0.08	−0.09
	p	0.36	0.61	0.74	0.85	0.20	0.71	0.50	0.30	0.21
Viability-24 h	r	−0.18^*^	−0.09	−0.05	− 0.08	−0.10	− 0.04	−0.03	0.10	−0.07
	p	0.01	0.21	0.48	0.30	0.16	0.57	0.64	0.18	0.32
SVI-24 h	r	−0.19^**^	−0.08	−0.06	− 0.09	−0.10	− 0.04	−0.05	0.09	−0.06
	p	0.01	0.26	0.40	0.24	0.17	0.56	0.51	0.20	0.39

*BMI* body mass index; *WC* waist circumference; *WHR* waist-to-hip ratio; *LDL-C* low density lipoprotein cholesterol; *HDL-C* high density lipoprotein cholesterol. *SST* sperm survival test. *PR* progressive; *SMI* sperm motility index; *SVI* sperm viability index

## Discussion

The effect of MetS on male fertility potential has been documented in several studies, reportedly, the semen volume, sperm concentration, progressive motility, and vitality, are significantly lower in individuals with MetS than in individuals without MetS [11–13]. Spermatozoa with abnormal mitochondrial membrane potentials, showing DNA fragmentation, have been observed in the context of MetS [21]. Meanwhile, other studies have reported that MetS does not seem to have a negative impact on semen parameters [14, 15]. MetS is considered as a condition of low-grade inflammation, including the inflammation of reproductive tract, in the absence of leukocytopenia [10]. Importantly, in this study, we showed that indeed, under certain conditions, MetS is associated with decreased sperm survival. The proportion of abnormal SST in the group with MetS was higher than that in the group without MetS.

Patients presenting with overweight/obesity are more likely to have abdominal fat storage, which potentially affects sperm quality. Adipokines (the cytokines produced by adipose tissue) may stimulate the production of reactive oxygen species (ROS) [22]; Further, obesity has also been associated with increased intestinal permeability and metabolic endotoxemia on male reproductive function, which then increase sperm DNA fragmentation and chromatin alterations [23]. Reportedly, increased scrotal adiposity is associated with testicular heat and oxidative stress, which negatively affect semen quality, sperm concentration, motility, and morphology [24, 25]. In this study, we observed that the prevalence of patients with MetS among infertile cases was higher than the general prevalence of MetS in Vietnamese [17] and Chinese [26] men. Comparing individuals with normal SST results with those with abnormal SST results showed that the WC was significantly higher in participants with abnormal SST, while the WHR was not. Therefore, in line with previous studies, our data strongly suggested that MetS, being overweight, and obesity are negatively associated with sperm survival.

Age is a factor that affects sperm survival. Advancement in age can lead to increased chromosomal breaks and point mutations in germ cells. Laurentino et al. identified a sharp increase in sperm DNA instability in males aged over 60 years [27]. Further, Moskovtsev et al., also reported that younger men showed a significantly lower decrease in SMI and SVI than men with 40 and above [6]. However, using conventional semen metrics to detect defects in spermatogenesis that are associated with increased DNA fragmentation in older men is challenging [28]. In this study, the mean age of participants in abnormal SST group was higher than that of the participants in the normal SST group; however, the difference was not statistically significant. This may be attributed to a selection bias; a relatively young population with a mean age of 34.7 ± 5.5 years was studied. Importantly, a remarkably weak negative correlation was observed between age and the SST parameters at 24 h, including the PR, SMI, vitality, and SVI. This is consistent with the results reported in the above mentioned previous studies.

Sperm preparation occurs in a medium that can improve the sperm viability. In the female tract, spermatozoa are protected from the harsh environment of the vagina before they swim up to the uterus through the cervical canal. Further, the cervical mucus filters out spermatozoa with poor morphology and motility, cellular fragments, round cells, blood cells. Thus, only a minority of ejaculated spermatozoa actually pass through the cervix [29, 30]. Additionally, the sperm preparation method was conducted in a similar manner to eliminate any unfavorable components that could produce toxic or free oxygen radicals, which can eventually affect sperm quality, especially sperm DNA integrity [31, 32]. Furthermore, given that initial sperm DNA damage has an effect on their subsequent survival [6], the density gradient sperm preparation method was used. With this method, it was possible to remove leukocytes and dead spermatozoa; thus, the method plays an important role in the consequent prevention of ROS generation.

Limitations to the study: as oligozoospermia was also included into this study population, the endocrine profile (FSH, LH, and Testosterone) as well as results of the genetic checkup (karyotype and AZF genes mutation) might play a role. Unfortunately, not all participants in this study had both hormonal tests and genetic screening. The remaining men were at the first steps of fertility assessment and we did not collect all the requested information. These missing data constitute a limitation to the study.

## Conclusion

In this study, we reported that men of infertile couples with MetS or abnormal WC show a higher rate of abnormal SST results. Although their SMI and SVI values at 24 h showed no significant differences compare with those of individuals without MetS, this was not the case for individuals with high BMI. In this specific group, men with MetS showed significantly higher occurrence of abnormal SST results. Importantly, weak negative correlations between the ages of the participants and several SST indicators were observed. Therefore, taken together, our data suggest that MetS, together with age and BMI, are independent factors associated with abnormal SST results.

## Data Availability

The dataset used or analyzed during the current study are available from the corresponding author upon reasonable request.

## Abbreviations

BMI: Body mass index
HDL-C: High-density lipoprotein cholesterol
IVF: In-vitro fertilization
LDL-C: Low-density lipoprotein cholesterol
MetS: Metabolic syndrome
NCEP ATP III: National cholesterol education program adult treatment panel III
PR: Progressive motility
ROS: Reactive oxygen species
SMI: Sperm motility index
SST: Sperm survival test
SVI: Sperm vitality index
TG: Triglycerides
WC: Waist circumference
WHO: World health organization
WHR: Waist-to-hip ratio
